# The RNA-binding protein HuR is a novel target of Pirh2 E3 ubiquitin ligase

**DOI:** 10.1038/s41419-021-03871-w

**Published:** 2021-06-05

**Authors:** Alexandra Daks, Alexey Petukhov, Olga Fedorova, Oleg Shuvalov, Alena Kizenko, Elizaveta Tananykina, Elena Vasileva, Oleg Semenov, Andrew Bottrill, Nickolai Barlev

**Affiliations:** 1 Institute of Cytology, Russian Academy of Sciences, 194064 St Petersburg, Russian Federation; 2 Almazov National Medical Research Centre, Institute of Hematology, 197341 St Petersburg, Russian Federation; 3 School of Life Sciences, University of Warwick, Coventry, CV4 7AL UK; 4 Moscow Institute of Physics and Technology, 141700 Dolgoprudny, Moscow Region Russian Federation

**Keywords:** Oncogene proteins, Ubiquitylation

## Abstract

The RING-finger protein Pirh2 is a p53 family-specific E3 ubiquitin ligase. Pirh2 also ubiquitinates several other important cellular factors and is involved in carcinogenesis. However, its functional role in other cellular processes is poorly understood. To address this question, we performed a proteomic search for novel interacting partners of Pirh2. Using the GST-pulldown approach combined with LC-MS/MS, we revealed 225 proteins that interacted with Pirh2. We found that, according to the GO description, a large group of Pirh2-associated proteins belonged to the RNA metabolism group. Importantly, one of the identified proteins from that group was an RNA-binding protein ELAVL1 (HuR), which is involved in the regulation of splicing and protein stability of several oncogenic proteins. We demonstrated that Pirh2 ubiquitinated the HuR protein facilitating its proteasome-mediated degradation in cells. Importantly, the Pirh2-mediated degradation of HuR occurred in response to heat shock, thereby affecting the survival rate of HeLa cells under elevated temperature. Functionally, Pirh2-mediated degradation of HuR augmented the level of c-Myc expression, whose RNA level is otherwise attenuated by HuR. Taken together, our data indicate that HuR is a new target of Pirh2 and this functional interaction contributes to the heat-shock response of cancer cells affecting their survival.

## Introduction

Pirh2 (p53-induced RING-H2 protein) was first described as an androgen receptor N-terminal interacting protein in 2002^1^. Human Pirh2 is coded by the *RCHY1* gene (RING-finger and CHY-zinc-finger domain-containing protein 1) and belongs to the RING-finger domain-containing proteins, many of which are E3 ligases. Accordingly, Pirh2 contains the centrally located RING domain responsible for the ubiquitin ligase activity, flanked by the N- and C-terminal domains responsible for protein–protein interactions^2^.

Pirh2 is a transcriptional target of the p53 tumor suppressor protein in mice. Similar to another p53-specific E3 ligase, Mdm2, Pirh2 is able to ubiqutinate p53 and target it for the proteasomal degradation^3^. Given the importance of the p53 for carcinogenesis, it is perhaps not surprising that Pirh2 is being studied mostly in the context of p53 suppression.

Besides p53, Pirh2 was shown to ubiquitinate two other members of the p53 protein family—p63 and p73^4–6^. Pirh2, unlike MDM2, is able to degrade active p53 under the conditions of DNA damage^7^, when p53 undergoes lysine-specific acetylation and methylation^8^. It is important to note that Pirh2 also mediates ubiquitination of mutant forms of the p53 protein, which are oncogenic, and the N-terminally truncated oncogenic isoform of p63 (ΔNp63)^9,10^. These data raise a question whether the cellular function of Pirh2 is oncogenic or tumor suppressive. To date, not much is known about the regulatory mechanisms of the *RCHY1* gene expression. There are only two transcription factors, p53 and its homolog p63, which were shown to control the *RCHY1* gene expression^3,11^.

Besides the p53 family members, Pirh2 is known to degrade a number of proteins playing key roles in such cellular processes as DNA damage response, cell cycle progression, gene expression regulation, and tumor transformation. These proteins include Polη, Chk2, p27^Kip1^, and HDAC1^12–15^.

Given the spectrum of Pirh2 targets, it is not surprising that Pirh2 is often implicated either as a biomarker and/or a prognostic factor in different types of cancer. In general, an elevated Pirh2 level is associated with tumor transformation, and a poor outcome and increased cancer aggressiveness. Accordingly, Pirh2 was shown to be upregulated in lung cancer^16^, prostate cancer^12^, and head and neck cancers^17^, and is associated with a poor prognosis of patients with glioma^18^, hepatocellular carcinoma^19^, and oral squamous cell carcinoma^20^. In contrast, the analysis of genome-wide microarray data revealed that lower levels of Pirh2 mRNA are associated with a reduced survival of patients with breast and ovarian cancer, and lung squamous carcinomas^21^. Thus, the role of Pirh2 in carcinogenesis is still debatable and requires further elucidation.

There are 86 Pirh2-interacting proteins known to date, including those identified only by high-throughput screenings without further validation. In drastic contrast, there are 522 known interacting partners for the p53 ubiquitin ligase, MDM2 (https://thebiogrid.org/), which clearly indicates that the interactome of Pirh2 is underestimated. To broaden our knowledge about the p53-independent role of Pirh2 in tumor transformation and other physiological processes, we performed a proteomic search for additional Pirh2-interacting partners. Using various experimental approaches, we demonstrated that Pirh2 was involved in the regulation of the RNA-binding protein, HuR1 (ELAVL1), thereby affecting the mRNA stability of its target genes, including *c-Myc*.

## Materials and methods

### Plasmids

The pcDNA-Pirh2 construct was obtained from Dr S. Benchimol. For expressing Pirh2-3×FLAG and HuR-3×FLAG, the pIRES-hrGFP-1a vector (Agilent Technologies, Santa Clara, CA, USA) was used. For expressing Pirh2-GST and HuR-GST in *Escherichia coli*, the corresponding inserts were cloned into the pGEX-5X-1 vector backbone (GE Healthcare, Milwaukee, WI, USA) and were subsequently purified on Glutathione 4B sepharose (GE Healthcare, Milwaukee, WI, USA). The Ku70-GST protein was obtained as described previously^22^. For ubiquitination assay experiments, the pсDNA-6His-Ubiquitin vector was used. An empty pcDNA3.1 vector was used as negative control. For stable Pirh2 knockdown (KD), the pLKO.1-TRC vector^23^ with a Pirh2-specific short hairpin RNA (shRNA) insert^24^, the lentiviral packaging plasmid psPAX2 (Addgene #12260), and the envelope plasmid pMD2.G (Addgene #12259) were used. For HuR KD, the following shRNA were used: ELAVL1_top 5′-CCGGCGAGCTCAGAGGTGATCAAAGCTCGAGCTTTGATCACCTCTGAGCTCGTTTTTG-3′ and ELAVL1_bot 5′-AATTCAAAAACGAGCTCAGAGGTGATCAAAGCTCGAGCTTTGATCACCTCTGAGCTCG-3′. For stable Pirh2 overexpression (Pirh2 OE), LeGO-iG2 vector^25^ was used.

### Cell cultures

All cell lines were cultured in standard conditions, using appropriate culture media supplemented with 10% fetal bovine serum (Walkersville, MD, USA), 100 units/ml penicillin, 100 mg/ml streptomycin, and 2 mM l-glutamine. Human embryonic kidney (HEK293T), human breast adenocarcinoma (MDA-MB-231), and human cervix adenocarcinoma (HeLa) cells were cultured in Dulbecco’s modified Eagle’s medium. For human non-small cell lung carcinoma (H1299) cells, RPMI 1640 medium was used. Cells were grown at 37 °C in 5% CO_2_ humidified atmosphere. H1299 cells were transfected using X-tremeGENE HP reagent (Sigma Aldrich, St. Louis, MO, USA) and HEK293T cells were transfected using TurboFect (Thermo Fisher Scientific, Waltham, MA, USA) according to the manufacturer’s instructions. H1299, HeLa, and MDA-MB-231 cell lines stably overexpressing Pirh2, and H1299 and HeLa cell lines with Pirh2 KD were obtained using the protocol described previously^24^. All cell lines were obtained from ATCC and were checked regularly for mycoplasma infections.

### GST-pulldown assay and mass spectrometry

Glutathione-*S*-transferase (GST)-pulldown assay was performed using the previously developed approach^26,27^. Briefly, the Pirh2-GST recombinant protein and an appropriate amount of GST were immobilized on glutation-sepharose beads (GE Healthcare, Milwaukee, WI, USA), which were then added to the cell lysate and incubated with rotation at 4 °C for 3 h. After washing with phosphate-buffered saline (PBS), retained proteins were eluted and subjected to either mass spectrometry (MS) analysis or western blotting for detection. The MS analysis was carried out as described previously^27^. Briefly, the proteins bound to GST or Pirh2-GST respectively were separated by SDS-polyacrylamide gel electrophoresis, excised from the gel, and subjected to trypsin digest in the gel. Liquid chromatography–tandem MS (LC-MS/MS) was carried out using an RSLCnano high-performance liquid chromatography system (Dionex, Sunnyvale, CA, USA) and an LTQ-Orbitrap-Velos mass spectrometer (Thermo Fisher Scientific, Waltham, MA, USA).

### Co-immunoprecipitation

The co-immunoprecipitation protocol was described previously^28^. Briefly, HEK293T cells were transiently transfected with an empty pIRES-hrGFP-1a vector and the vector coding Pirh2-3×FLAG. Forty-eight hours after transfection, the cells were collected for lysates preparation with a modified radioimmunoprecipitation assay buffer. The lysates were then incubated with anti-FLAG M2 agarose beads (Sigma Aldrich, St. Louis, MO, USA) for 4 h at 4 °C with rotation. Following washes, the bound proteins were eluted by a 3× FLAG peptide solution and analyzed by western blotting.

Co-immunoprecipitation of endogenous Pirh2 and HuR proteins was carried out in extract made from 1 × 10^7^ HeLa cells essentially as described in ref. ^29^. Two equal portions of cell lysate were incubated with 5 μg anti-HuR mouse monoclonal antibodies and equal amount mouse IgG as a negative control at 4 °C for 4 h with rotation. Protein G agarose magnetic beads (Thermo Fisher Scientific, Waltham, MA, USA) was pre-incubated with 0.1% bovine serum albumin for 1 h to minimize the unspecific binding. Then, pre-incubated Protein G agarose was added to cell lysates containing antibodies and incubated at 4 °C for 1 h with rotation. After the incubation, the beads were washed three times with ice-cold lysis buffer using a magnet. The bound proteins were analyzed by western blotting using temperature-denaturated co-IP samples in Laemmli buffer.

### Ubiquitination assay

In cellulo ubiquitination was performed as previously described^30^. HEK293T cells were transiently transfected with pIRES-HuR, pcDNA-6His-Ubiquitin, and pcDNA-Pirh2 vectors. An empty pcDNA3.1 plasmid was used as negative control. Twenty-four hours after transfection, cells were treated with 10 μM MG132 for 16 h and then lysed in 8 M urea-containing buffer (pH 8.0). 6His-ubiquitinated proteins were purified on Ni-NTA beads (Qiagen, Hilden, Germany). The bound proteins were eluted and analyzed by western blotting.

In vitro ubiquitination assay was performed using Ubiquitinylation kit (BML-UW9920; Enzo Biochem, New York, NY, USA) according to the manufacturer’s recommendations. The purified GST-tagged proteins were used for in vitro ubiquitination reaction: HuR-GST was used as a substrate; full-length Pirh2 (Pirh2 FL-GST), N-terminal domain of Pirh2 (Pirh2 NTD-GST), and GST as the negative control were used for their E3 in vitro ligase activity investigation. The protein ratios used for the reaction are demonstrated in Supplementary Fig. S5.

### RNA immunoprecipitation

To detect RNAs associated with the HuR protein, RNA immunoprecipitation (RIP) was performed. H1299 cells (8 × 10^6^) were collected by trypsinization and resuspended in ice-cold RIP buffer containing 150 mM KCl, 25 mM Tris-HCl pH 7.4, 5 mM EDTA, 0.5 mM dithiothreitol, 0.5% NP-40, 100 μg/ml RNAse inhibitor RiboLock (Thermo Fisher Scientific, Waltham, MA, USA), and protease inhibitor cocktail (Sigma Aldrich). After 10 min incubation in the RIP buffer and centrifugation at 13,000 × *g* for 5 min, supernatant was collected and used for subsequent manipulations. Seven micrograms of anti-HUR antibodies (3A2, Santa Cruz Biotechnology, Santa Cruz, CA, USA) were added to the supernatant and incubated for 4 h at 4 °C followed by incubation with 40 μl of protein G magnetic beads (Thermo Fisher Scientific, Waltham, MA, USA) per sample for 1 h at 4 °C with rotation. After the incubation, the beads were washed three times with ice-cold RIP buffer using a magnet and then RNA was isolated by Trizol reagent (Thermo Fisher Scientific, Waltham, MA, USA) according to the manufacturer’s instruction. Total RNA calculated according to the number of cells was used as input control. To analyze the immunoprecipitated mRNA, reverse-transcriptase PCR (RT-PCR) was performed. cDNA was synthetized using Reverse Transcription Kit (Thermo Fisher Scientific, USA) according to the manufacturer’s recommendations. Quantitative PCR (qPCR) was performed using primers for coding sequence (CDS) and 3′-untranslated region (3′-UTR) of the c-Myc mRNA sequence. The following primers were used: c-Myc CDS Fwd-5′-CTCCTCCTCGTCGCAGTAGA-3′, c-Myc CDS Rev-5′-GCTGCTTAGACGCTGGATTT-3′; c-Myc 3′-UTR Fwd-5′-AACCTTGGCTGAGTCTTGAG-3′, Rev-5′-AGTTCTTTTATGCCCAAAGTCCA-3′. Glyceraldehyde 3-phosphate dehydrogenase (GAPDH) signal was used as a reference and normalization control.

### Colony-formation assay

The assay was performed essentially as described previously^24^. Five hundred cells, both treated at 45 °C and untreated, were seeded in RPMI 1640 medium on 30 mm plates and incubated for 7 days for colony formation. The colonies were fixed and stained for 10 min with fixing/staining solution containing 0.05% crystal violet, 1% formaldehyde, and 1% methanol buffered with PBS. Following washes and drying, the colonies were scored and analyzed manually. The experiments were performed in triplicates.

### Cell cycle analysis

Cell cycle analysis was performed as previously described^31^. Briefly, collected cells were washed twice with PBS followed by incubation with 1% saponin for 20 min. Then, DNA was treated by 1 mg/ml RNase A and stained with 50 mg/ml propidium iodide for 30 min. Flow cytometry was performed using the CytoFLEX instrument (Beckman Coulter, Inc., Miami, FL, USA). Analysis was carried out using CytExpert Software.

### Real-time cell proliferation assay

These tests were performed using the xCELLigence system (ACEA Biosciences, San Diego, CA, USA) according to the manufacturer’s instruction. For cell proliferation assays, 2 × 10^4^ cells were seeded in each well of E-plate 16 (ACEA Biosciences, San Diego, CA, USA) in RPMI 1640 medium. Cell index was registered every 15 min.

### Real-time PCR

To assess the mRNA levels, total RNA was extracted from cells using Trizol reagent (Sigma Aldrich, St. Louis, MO, USA). cDNA was synthetized using Reverse Transcription Kit (Thermo Fisher Scientific, Waltham, MA, USA) according to the manufacturer’s recommendations. qPCR was performed after cDNA synthesis using previously published primers^24,28^. For HuR analysis, the following primers were used: forward 5′-GAGGCTCCAGTCAAAAACCA-3′ and reverse 5′-GTTGGCGTCTTTGATCACCT-3′. mRNA expression levels were calculated relative to GAPDH by ∆∆Ct method.

### Western blotting

For western blot analysis (WB), whole-cell extracts were prepared unless otherwise specified. The primary antibodies against the analyzed proteins were used as follows: Pirh2 (EPR14980, Abcam), β-actin (A3854, Sigma Aldrich, St. Louis, MO, USA), c-Myc (9402S, Cell Signaling Technology, Beverly, MA, USA), FLAG (F1804, Sigma Aldrich, St. Louis, MO, USA), HuR (12582S, Cell Signaling Technology, Beverly, MA, USA), β-tubulin (T8535, Sigma Aldrich, St. Louis, MO, USA), and His-Tag (2365, Cell Signaling Technology, Beverly, MA, USA); secondary antibodies used were anti-mouse and anti-rabbit (Sigma Aldrich, St. Louis, MO, USA).

### Bioinformatics

Correlations of expression levels of *RCHY1* (Pirh2) and *myc* (c-Myc) with the survival rates of lung cancer patients were calculated by algorithms described by Amelio et al.^32^. The individual contribution of *ELAVL1* (HuR) was examined as described in Antonov et al.^33^. using the Gene Expression Omnibus (GEO) microarray data.

### Statistical analysis

Data are shown as mean ± SD or SEM of at least three replicates. Statistical significance was analyzed using Student’s *t*-test. *P* < 0.05 was considered significant. *P* < 0.01 is denoted as an asterisk “*”.

## Results

### Identification of novel Pirh2-interacting proteins

**Fig. 1:**
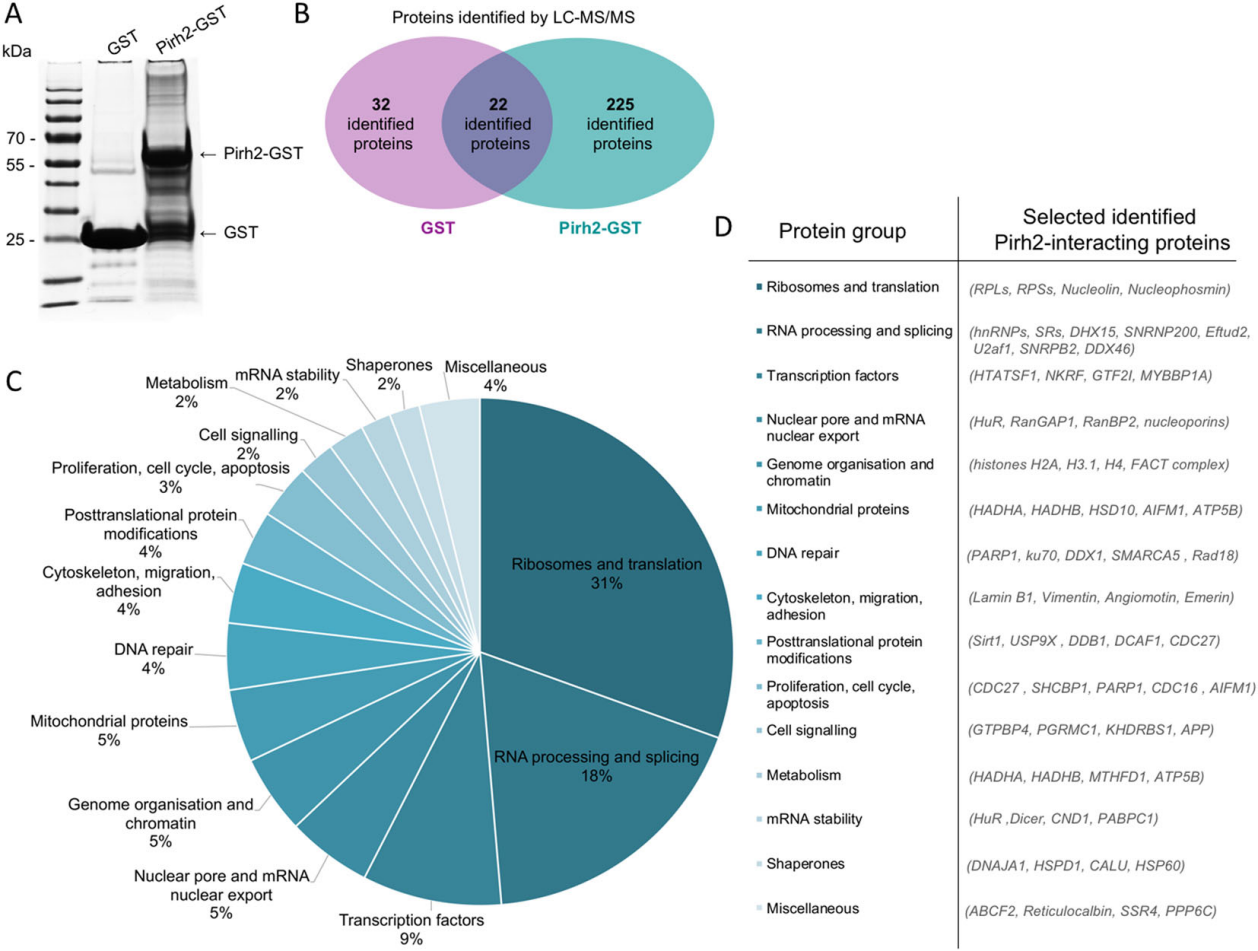
Interactome of Pirh2. **A** Coomassie-stained gel of GST and Pirh2-GST proteins after incubation with cell extracts from HEK293T. Pirh2-GST fusion protein and native GST protein are denoted with arrows. The mass spectrometry data were processed using Mascot49 and Scaffold 4 software. **B** Euler diagram demonstrating the number of GST- and Pirh2-GST-interacting proteins identified by LC-MS/MS. **C** A pie chart of identified Pirh2-interacting proteins grouped by their functions. **D** The list of functionally distinguished groups with selected representative proteins that interact with Pirh2.

To identify the proteins that interact with Pirh2 and, hence, may represent its potential targets for ubiquitination, we used the GST-pulldown approach combined with high-resolution MS (Fig. 1). To this end, the recombinant protein Pirh2-GST was incubated with the cell extract prepared from the human embryonic kidney cell line HEK293T (Fig. 1A). Native GST protein was used as control for nonspecific binding. The Pirh2-GST-bound proteins were identified by LC-MS/MS.

The MS analysis has yielded 346 interacting proteins in total: 50 proteins interacted with GST only, 256 proteins interacted specifically with Pirh2-GST, and 40 proteins interacted with both GST and Pirh2-GST. The raw data were published in the Mendeley Datasets Repository (10.17632/24bh4cvhzd.1). The data processing consisted of excluding prokaryotic proteins, keratins, Pirh2 protein, and GST from the list of interacting proteins. Each identified protein, for which at least three peptides were detected (score ≥ 3), and which either did not bind or bound poorly GST compared to Pirh2-GST, was defined as significant. After processing the data obtained, we revealed 225 proteins bound to Pirh2, but not GST (Fig. 1B and Supplementary Table S1).

We grouped the identified proteins according to their functions in the cell. As a result, we found that almost half of the identified proteins belong to “Ribosomes and translation” and “RNA processing and splicing” groups (Fig. 1C, D). In total, we combined all identified proteins into 15 groups, with several representative proteins from each group being shown in Fig. 1C, D. Among the Pirh2-interacting proteins were the ones that are involved in gene expression regulation, DNA repair, apoptosis, and tumor transformation (e.g., PARP1, ku70, Sirt1, HuR, and Dicer).

To validate our GST-pulldown results, we chose one of the highest (HuR) and one of the lowest scoring proteins (Ku70), and carried out reciprocal pulldowns of Pirh2 using the Ku70-GST or HuR-GST recombinant proteins. Both HuR-GST and Ku70-GST, but not GST alone, were able to bind Pirh2 (Supplementary Fig. S1). Thus, we confirmed the specificity of protein–protein interactions with Pirh2 in our assay.

### Pirh2 interacts with and ubiquitinates the RNA-binding protein HuR leading to its proteasomal degradation

**Fig. 2:**
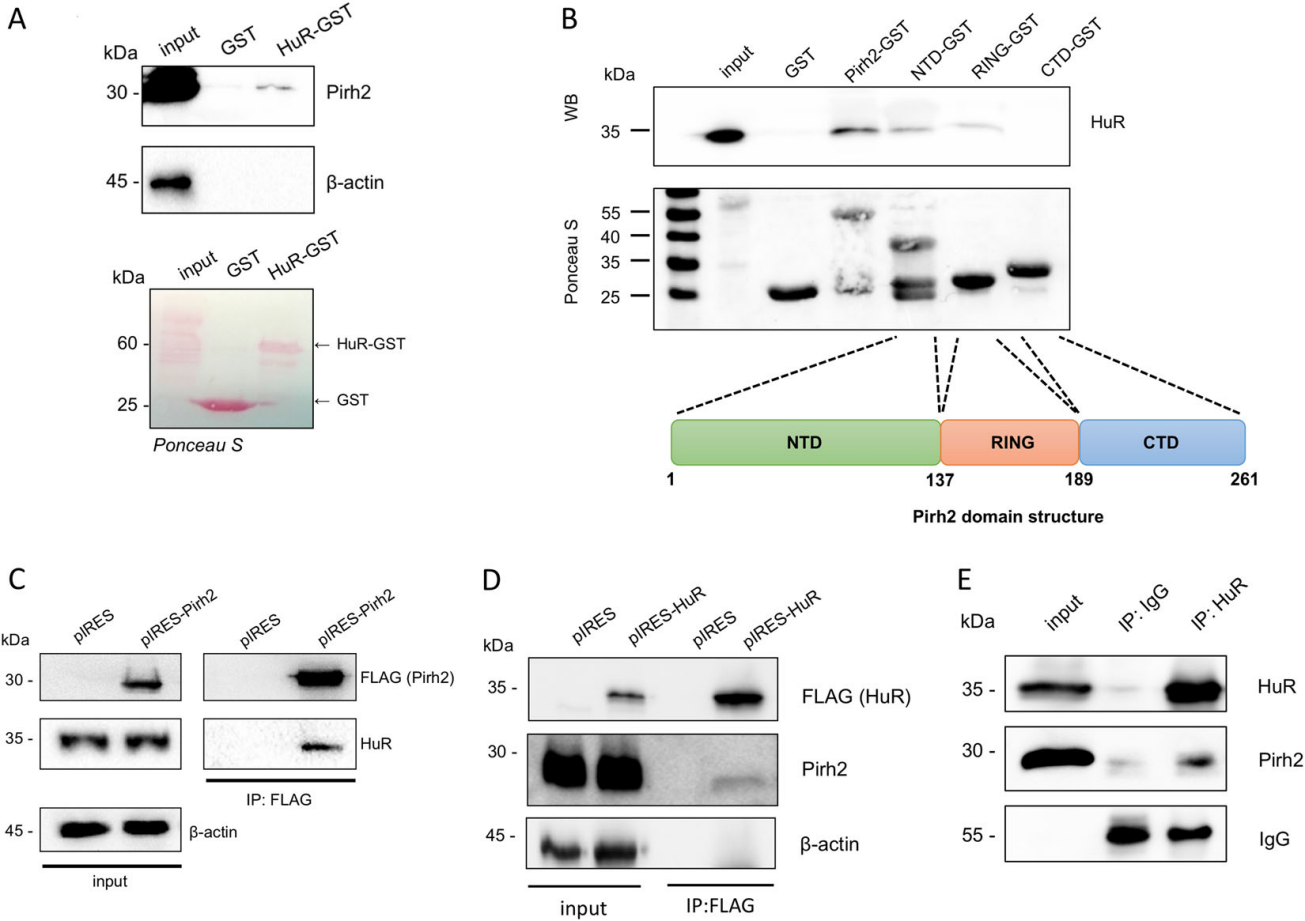
Pirh2 binds to HuR in vitro and in cellulo. **A** HuR-GST interacts with Pirh2. GST-pulldown assay with extracts from HEK293T, demonstrating the interaction of HuR-GST with Pirh2. A Ponceau-stained membrane. HuR-GST fusion protein and native GST protein are denoted with arrows (left); western blot analysis of Pirh2 binding to GST and HuR-GST (right). **B** Identification of the Pirh2 domains involved in HuR binding. WB analysis of GSTpull-down assay with purified GST-tagged proteins: GST, full-length Pirh2 (Pirh2-GST), the N-terminal domain of Pirh2 1–132 a.a. (NTD-GST), the RING domain of Pirh2 138–189 a.a. (RING-GST), and the C-terminal domain of Pirh2 190–261 a.a. (CTD-GST) (upper panel); the scheme demonstrating the Pirh2 domain structure (bottom panel). **C** Pirh2 interacts with HuR in cellulo. Western blot analysis of co-immunoprecipitation in HEK293T cells of ectopically expressed Pirh2-3×FLAG (pIRES-Pirh2) and endogenous HuR. An empty pIRES vector was used as negative control. **D** HuR interacts with Pirh2 in cellulo. Western blot analysis of co-immunoprecipitation in HeLa cells of ectopically expressed HuR-3×FLAG (pIRES-HuR) and endogenous Pirh2. An empty pIRES vector was used as negative control. **E** Co-immunoprecipitation of the endogenous HuR and Pirh2 proteins using anti-HuR monoclonal antibodies.

As our results clearly suggested that Pirh2 is important for the expression of proteins, we questioned whether Pirh2 can regulate the mRNA stability, thereby indirectly affecting the protein biosynthesis. It is noteworthy that HuR, a global regulator of mRNA stability, has emerged as one of the strong Pirh2 interactors (Fig. 2). Thus, we decided to investigate the functional significance of this interaction. First, we performed reciprocal GST-pulldown assay using the purified HuR-GST protein and the cellular extract prepared from HEK293T cells. We demonstrated that HuR-GST binds the endogenous Pirh2 protein more strongly compared to GST, supporting the previously obtained LC-MS/MS data (Fig. 2A).

To determine the portion of Pirh2 responsible for HuR binding, we employed GST-pulldown assay. To this end, purified GST-tagged deletion mutants of Pirh2 (the N-terminal domain, 1–137 a.a. (NTD-GST), the RING domain, 138–189 a.a. (RING-GST), and the C-terminal domain, 190–261 a.a. (CTD-GST)) were incubated with the whole-cell extract from HEK293T. The full-length GST-Pirh2 protein and GST alone were also analyzed as positive and negative controls, respectively. Results of the binding experiment (Fig. 2B) clearly suggest that the N-terminal and RING domains of Pirh2 are responsible for the Pirh2-HuR interaction.

To confirm the GST-pulldown interaction results, we performed reciprocal co-immunoprecipitation of endogenously expressed HuR and ectopic Pirh2-3×FLAG or, conversely, endogenously expressed Pirh2 and ectopic HuR-3×FLAG. We found that that Pirh2 specifically interacted with HuR in cellulo (Fig. 2C, D).

We also confirmed the Pirh2-HuR interaction on the endogenous level by co-immunoprecipitation. The endogenous Pirh2-HuR complex from HeLa cells was precipitated using anti-HuR monoclonal antibodies (Fig. 2E). Taken together, these results confirm an existence of the Pirh2-HuR interaction in cells.

**Fig. 3:**
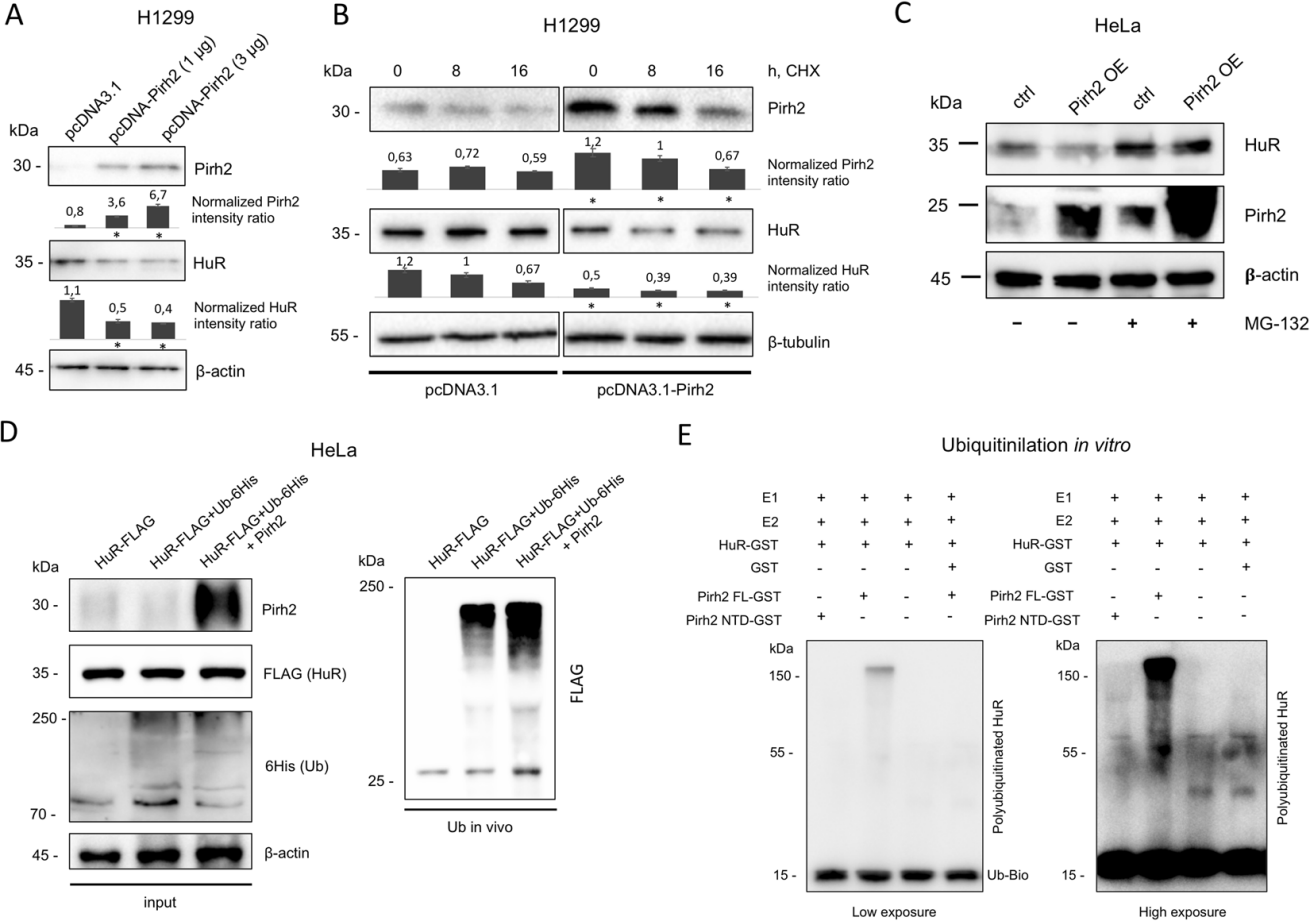
Pirh2 downregulates HuR level via ubiquitination and targeting for the degradation. **A** Pirh2 decreases HuR level in H1299 cells. Transfection of H1299 cells with different quantity of pcDNA3-Pirh2 plasmid proportionally decreases HuR level. Empty pcDNA3.1 was used as a control and to keep the total plasmid DNA amount 3 μg in each sample. The normalized intensity ratios were calculated as ratios between the signals of proteins analyzed and the corresponding actin bands on the basis of three measurements. Error bars indicate ± SD. *p ≤ 0.05 vs. empty vector transfection according to Student’s *t*-test. **B** Pirh2 shortens half-life time of HuR. Western blot analysis of HuR protein level in H1299 cells transfected with pcDNA-Pirh2 and pcDNA3.1 empty vector, and treated with 50 μM cycloheximide (CHX) for 8 and 16 h. The normalized intensity ratios were calculated as ratios between the signals of proteins analyzed and the corresponding actin bands on the basis of three measurements. Error bars indicate ±SD. *p ≤ 0.05 vs. empty vector transfection for the corresponding time points according to Student’s *t*-test. **C** WB analysis of HuR level in HeLa cells transiently transfected with pcDNA3-Pirh2 plasmid (Pirh2 OE) and pcDNA3.1 as a control (ctrl). The cells were treated with 10 μM MG132 for 16 h. **D** Pirh2 ubiquitinates HuR in cellulo. Western blot analysis of Ni-agarose-precipitated 6His-ubiquitinated HuR protein from HeLa cells transfected with pIRES-HuR (HuR-3×FLAG); pIRES-HuR and pcDNA-6His-Ubiquitin (Ub-6His); and pIRES-HuR, pcDNA-6His-Ubiquitin, and pcDNA-Pirh2 (Pirh2). pcDNA3.1 vector was used to keep the same amount of total plasmid DNA in each sample (right). Western blot analysis of Pirh2 and HuR-3×FLAG in inputs (left). **E** In vitro ubiquitinilation assay. Ubiquitin-activating enzyme (E1), UbcH5b (E2) and GST-tagged HuR (HuR-GST, substrate), and biotin-tagged ubiquitin were added in equal amounts in each portion of the reaction. GST, full-length Pirh2 (Pirh2 FL-GST), and the catalytically inactive N-terminal domain of Pirh2 1–132 a.a. (NTD-GST) were tested for their ability to ubiquitinate HuR. No E3 ligase control was also used. The WB analysis of the ubiquitinilation reaction was performed using anti-biotin antibodies. Two variants of exposure are shown for better illustration. The relative amounts of purified proteins used in the reaction are demonstrated in Supplementary Fig. S5.

Next, we analyzed whether Pirh2 affects the HuR protein level in cells. To test this, we transfected H1299 cells with increasing amounts of the Pirh2-expressing plasmid. The total amount of DNA in each sample was normalized with an empty pcDNA3.1 plasmid. As shown in Fig. 3A, increasing levels of Pirh2 expression led to a concomitant reduction of the HuR level. We also tested the effect of Pirh2 on HuR in another tumor cell line, MDA-MB-231, which constitutively expressed either control plasmid (LEGO) or Pirh2 (LEGO-Pirh2). Expectedly, constitutive overexpression of Pirh2 decreased the protein level of HuR (Supplementary Fig. S2).

To investigate whether the effect of Pirh2 on HuR involved its protein stability, we performed the cycloheximide-mediated blockade of the protein synthesis. To this end, H1299 cells transfected with the Pirh2-coding plasmid or the empty vector were used. As shown on Fig. 3B, ectopic expression of Pirh2 attenuated the level of HuR expression and ultimately reduced the half-life of the protein from 16 to 8 h. Thus, we confirmed that Pirh2 affected the protein level of HuR in different cell types. The fact that adding a proteasome inhibitor MG132 to HeLa cells stabilized the protein level of HuR even in the presence of Pirh2 indicated that HuR attenuation likely involved the ubiquitin-dependent proteasome system (Fig. 3C).

As Pirh2 is the E3 ubiquitin ligase that polyubiquitinates its targets leading to their proteasomal degradation, we assumed that HuR may be a novel substrate for Pirh2-dependent ubiquitination. To test this hypothesis, we performed ubiquitination of HuR in HeLa cells expressing 6His-ubiquitin and different levels of Pirh2 (Fig. 3D). Ectopic overexpression of Pirh2 increased the level of polyubiquitinated HuR, hence suggesting that HuR is a potential target of Pirh2-dependent ubiquitination. We also repeated the ubiquitination assay in HEK293T cells with essentially similar results, i.e., Pirh2 was able to ubiquitinated HuR (Supplementary Fig. S3). To address the question of Pirh2 involvement in ubiquitination of HuR, we also compared the efficacies of in cellulo ubiquitination of the endogenous HuR protein in the presence of the full-length or mutant Pirh2 (Pirh2 NTD) proteins (Supplementary Fig. S4A). Indeed, the full-length Pirh2 protein ubiquitinated HuR more efficiently compared to the Pirh2 NTD mutant protein. We also confirmed the inability of catalytically inactive mutant of Pirh2 to downregulate the HuR protein level (Supplementary Fig. S4B).

Furthermore, to prove that Pirh2 is involved in direct ubiquitination of HuR, we performed in vitro ubiquitination assay using purified GST-tagged full-length Pirh2 (Pirh2 FL-GST) and the catalytically inactive Pirh2 mutant (Pirh2 NTD-GST). We also used a non-fused GST protein as a negative control. As shown in Fig. 3E, efficient ubiquitination of HuR occurs only in the presence of the full-length Pirh2 protein.

Thus, we demonstrated that Pirh2 was able to interact and ubiquitinate the RNA-binding protein HuR leading to its destabilization on the protein level.

### Pirh2 affects HuR stability during the heat-shock response

It is well established that HuR protein plays a role in heat-shock response affecting the mRNA stability and cellular localization of its target genes, including mRNA of HSP70^34–36^. In addition, HuR was shown to form cytoplasmic stress granules in response to heat shock (HS), yet its total level in the cell decreased^37^. Therefore, to assess the physiological significance of Pirh2-mediated attenuation of HuR, we examined how Pirh2 affected the stability of HuR under the heat-shock stress conditions.

**Fig. 4:**
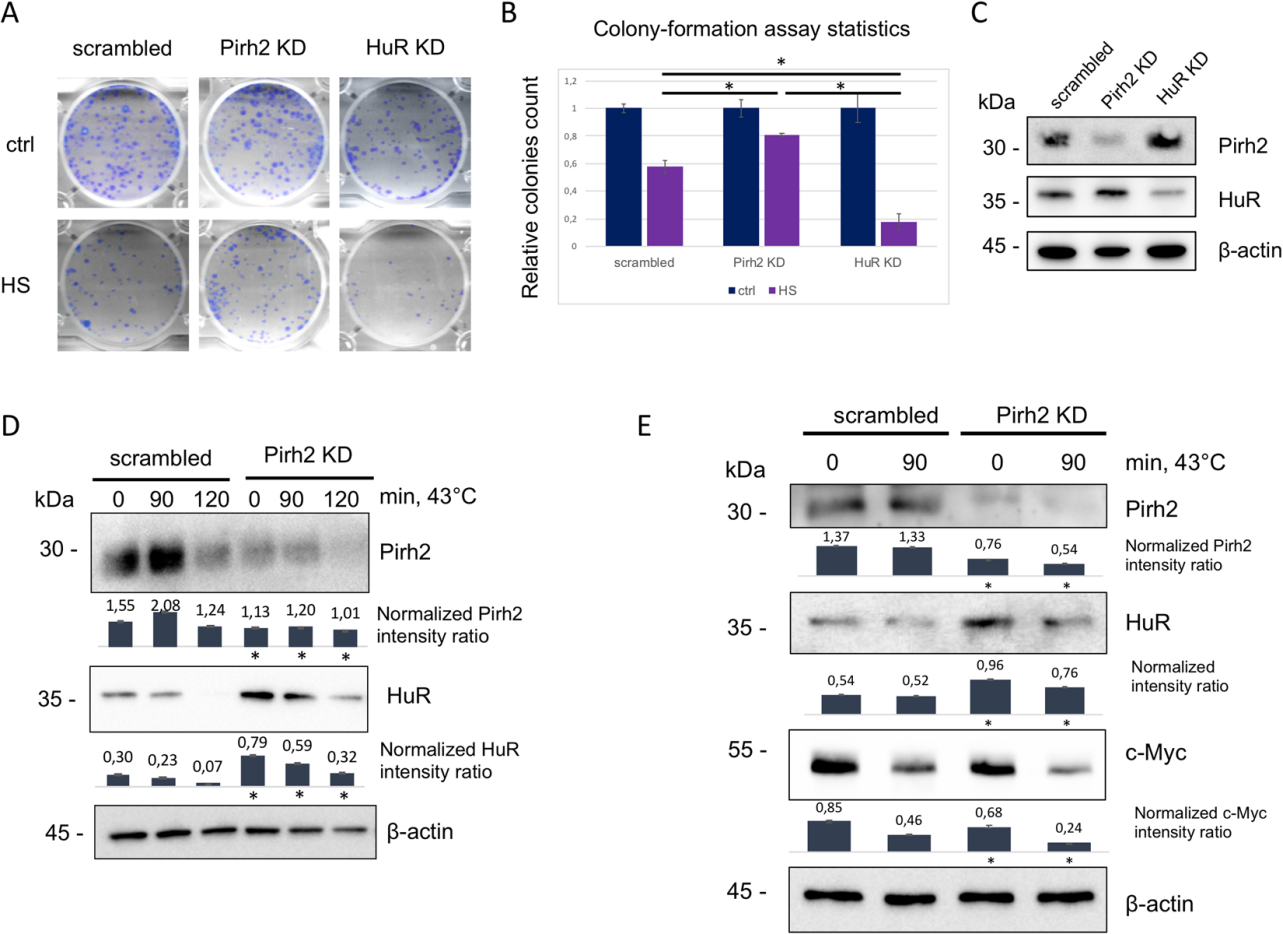
Pirh2 affects HuR stability, cellular survival, and cell cycle in HeLa cells during heat-shock response. **A** Knockdown of Pirh2 leads to an increase of HeLa cell viability after heat-shock exposition. Colony-formation assay of Pirh2 knockdown (Pirh2 KD), HuR knockdown (HuR KD), and control (scrambled) HeLa cells in normal condition (ctrl) and after 45 °C incubation for 3 h (HS). The test was performed in triplicates. **B** Statistical analysis of colony-formation assay. Error bars indicate ±SEM. **C** Western blot analysis demonstrating protein levels of Pirh2 and HuR in Pirh2 knockdown (Pirh2 KD), HuR knockdown (HuR KD), and control (scrambled) HeLa cells. **D** Pirh2 destabilizes HuR under heat shock. Western blot analysis of HuR level in HeLa cells with Pirh2 knockdown (Pirh2 KD) and control HeLa cells (scrambled) after incubating at 43 °C for 90 and 120 min. The normalized intensity ratios were calculated as ratios between the signals of proteins analyzed and the corresponding actin bands on the basis of three measurements. Error bars indicate ±SD. *p ≤ 0.05 vs. scrambled for the corresponding time points according to Student’s *t*-test. **E** Pirh2 stabilizes c-Myc protein in HeLa cells. Western blot analysis of Pirh2, HuR, and c-Myc levels in HeLa cells with Pirh2 knockdown (Pirh2 KD) and control HeLa cells (scrambled), both in normal condition and after incubating at 43 °C for 90 min. The normalized intensity ratios were calculated as ratios between the signals of proteins analyzed and the corresponding actin bands on the basis of three measurements. Error bars indicate ±SD. *p ≤ 0.05 vs. scrambled for the corresponding time points according to Student’s *t*-test.

We asked a question of whether Pirh2 via attenuating the HuR stability may affect cell survival after the treatment with sublethal HS. We performed colony-formation assay to test the viability of HeLa cells with suppressed Pirh2 or HuR expression compared to control cells after 45 °C heat-shock exposure for 3 h. Pirh2 KD led to a statistically significant increase in the number of colony-forming units compared to control cells, whereas KD of HuR had the opposite effect (Fig. 4A, B). The efficiency of Pirh2 and HuR KDs is demonstrated in Fig. 4C. We have also performed cell cycle analysis of HeLa cells with Pirh2 and HuR KD exposed to HS(45 °C, 2 h) followed by 16 h of recovery. In response to the heat-shock treatment, the amount of Pirh2 KD cells in the G2 phase was higher compared to control cells, whereas HuR KD cells displayed the opposite effect (Supplementary Fig. S6). As the sublethal dose of HS causes proteotoxic stress that mainly results in the G2/M cell cycle arrest^38,39^, we concluded that KD of Pirh2, which leads to an increased G2 arrest, correlated with cell survival.

Next, we investigated whether Pirh2 attenuated the level of HuR upon HS in HeLa cells. As shown in Fig. 4D, protein levels of both HuR and Pirh2 decreased upon the exposure of cells to high temperature. However, in HeLa cells with KD Pirh2, the level of HuR was higher at all the time points compared to the matching control cells. To address the question of whether KD of Pirh2 augments the amount of cellular HuR by increasing its mRNA level, we performed qRT-PCR analysis (Supplementary Fig. S7). We established that suppression of Pirh2 did not affect the HuR mRNA level. Noteworthy, in contrast to the expectation, attenuation of Pirh2 led to a moderate decrease of the HuR mRNA level, therefore strongly suggesting that the increased level of HuR observed in Pirh2 KD cells was due to the protein stabilization and not to the regulation of its transcription (Supplementary Fig. S7).

It has been reported that c-Myc proto-oncogene sensitizes cells to heat-induced cell death^40,41^. Hence, downregulation of c-Myc would be a plausible mechanism responsible for the survival of HeLa cells upon HS. In this respect, we have recently shown that Pirh2 was a positive regulator of c-Myc expression in H1299 cells, and that the attenuation of Pirh2 resulted in a decrease of c-Myc levels^24^. Notably, HuR is a negative regulator of c-Myc expression at the level of mRNA stability^42^. Taking these facts together, we hypothesized the existence of a putative regulatory loop, whereby Pirh2 stabilized c-Myc via the degradation of HuR, and the latter, in turn, inhibited the translation of c-Myc. To test this hypothesis, we ascertained whether Pirh2 KD simultaneously stabilized HuR and attenuated c-Myc, both in normal conditions and after HS. In agreement with our hypothesis, in Pirh2 KD cells we observed a modest, but reproducible, elevation of HuR levels concomitant with a decrease of the c-Myc level (Fig. 4E).

We also confirmed the involvement of c-Myc in the Pirh2-regulated heat-shock response. To this end, HeLa Pirh2 KD and scrambled control cells were transiently transfected with the c-Myc expression vector and the colony-formation abilities of these cell lines were compared before and after HS. In agreement with our hypothesis that the Pirh2/HuR interaction affects the HS response via c-Myc, we found that the forced expression of c-Myc sensitized HeLa cells to HS irrespective of the Pirh2 status (Supplementary Fig. S8).

### Pirh2 activates c-Myc expression via downregulation of HuR

**Fig. 5:**
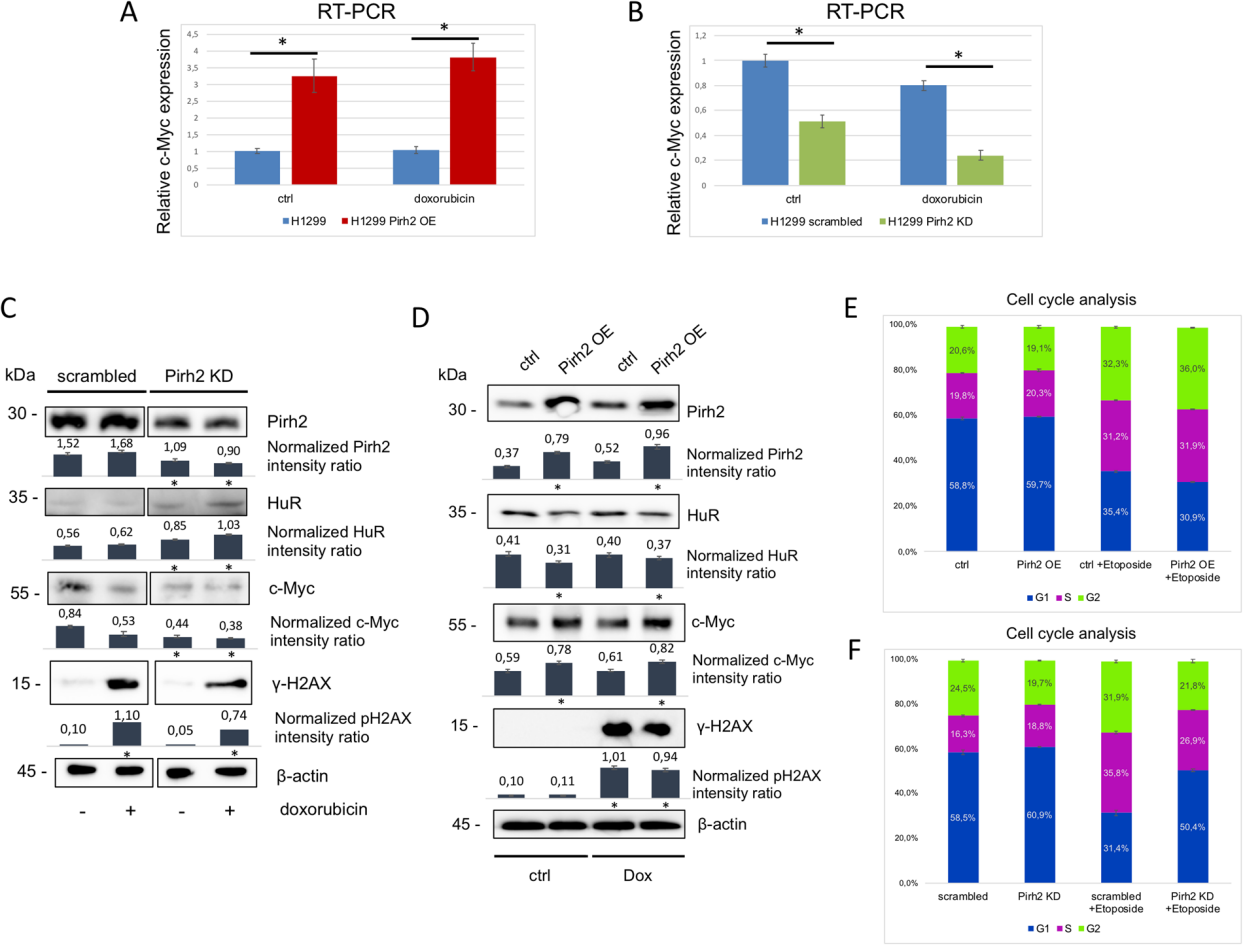
Pirh2 activates c-Myc expression via downregulation of HuR. **A** Pirh2 upregulates c-Myc mRNA level in H1299 cells. qRT-PCR results of c-Myc expression for H1299 cells with stable Pirh2 overexpression (Pirh2 OE) and control H1299 cells (ctrl), both in normal conditions and after 16 h treatment with 1 μM doxorubicin. *p ≤ 0.01 according to Student’s *t*-test. **B** Pirh2 knockdown leads to a decrease of c-Myc mRNA level. qRT-PCR results of c-Myc expression for H1299 cells with stable Pirh2 knockdown (Pirh2 KD) and control H1299 cells (scrambled), both in normal conditions and after 16 h treatment with 1 μM doxorubicin. *p ≤ 0.01 according to Student’s *t*-test. **C** Pirh2 upregulates c-Myc protein level in H1299 cells. Western blot analysis of c-Myc protein level for H1299 cells with stable Pirh2 overexpression (Pirh2 OE) and control H1299 cells (ctrl), both in normal conditions and after 16 h treatment with 1 μM doxorubicin. **D** Pirh2 knockdown leads to a decrease of c-Myc protein level. Western blot analysis of c-Myc level in H1299 cells with stable Pirh2 knockdown (Pirh2 KD) and control H1299 cells (scrambled), both in normal conditions and after 16 h treatment with 1 μM doxorubicin. The normalized intensity ratios were calculated as ratios between the signals of proteins analyzed and the corresponding actin bands on the basis of three measurements. Error bars indicate ±SD. *p ≤ 0.05 vs. scrambled (vs. untreated for γH2AX) according to Student’s *t*-test. **E**, **F** Analysis of cell cycle phases distribution for H1299 cell lines with stable Pirh2 overexpression (Pirh2 OE) and control H1299 (ctrl) (left panel), and H1299 cells with stable Pirh2 knockdown (Pirh2 KD) and control H1299 cells (scrambled) (right panel), both in normal conditions and after 24 h treatment with 100 μM etoposide. The test was performed in triplicates. Error bars indicate ±SD. *p-value ≤ 0.01 according to Student’s *t*-test.

As HuR was shown to downregulate c-Myc via recruiting the RNA miRNA-induced silencing complex (RISC) to its mRNA^42^, we first examined the effect of Pirh2 on c-Myc, on the level of mRNA in H1299 cells. When mRNA levels of c-Myc were compared between H1299 cells with constitutive Pirh2 OE and control cells (ctrl), it became apparent that the elevated c-Myc mRNA level correlated with overexpression of Pirh2 (Fig. 5A). On the contrary, the c-Myc mRNA level was attenuated in shRNA-mediated KD of Pirh2 (Pirh2 KD) (Fig. 5B). Interestingly, genotoxic stress caused by 1 μM doxorubicin treatment for 16 h had no effect on the ability of Pirh2 to stabilize the level of c-Myc as judged by RT-PCR (compare Fig. 5A, B). The same pattern was observed on the protein level (Fig. 5C, D).

Considering that c-Myc is a potent cell cycle regulator, we performed cell cycle analysis using isogenic cell lines that differed in the Pirh2 status. To induce genotoxic stress, cells were treated with Etoposide for the indicated time period. Pirh2-mediated G2/M block was observed in cells treated with 100 μM etoposide for 24 h (Fig. 5E). On the contrary, Pirh2 KD cells demonstrated the opposite effect (Fig. 5F).

### Pirh2 prevents HuR binding to c-Myc mRNA

**Fig. 6:**
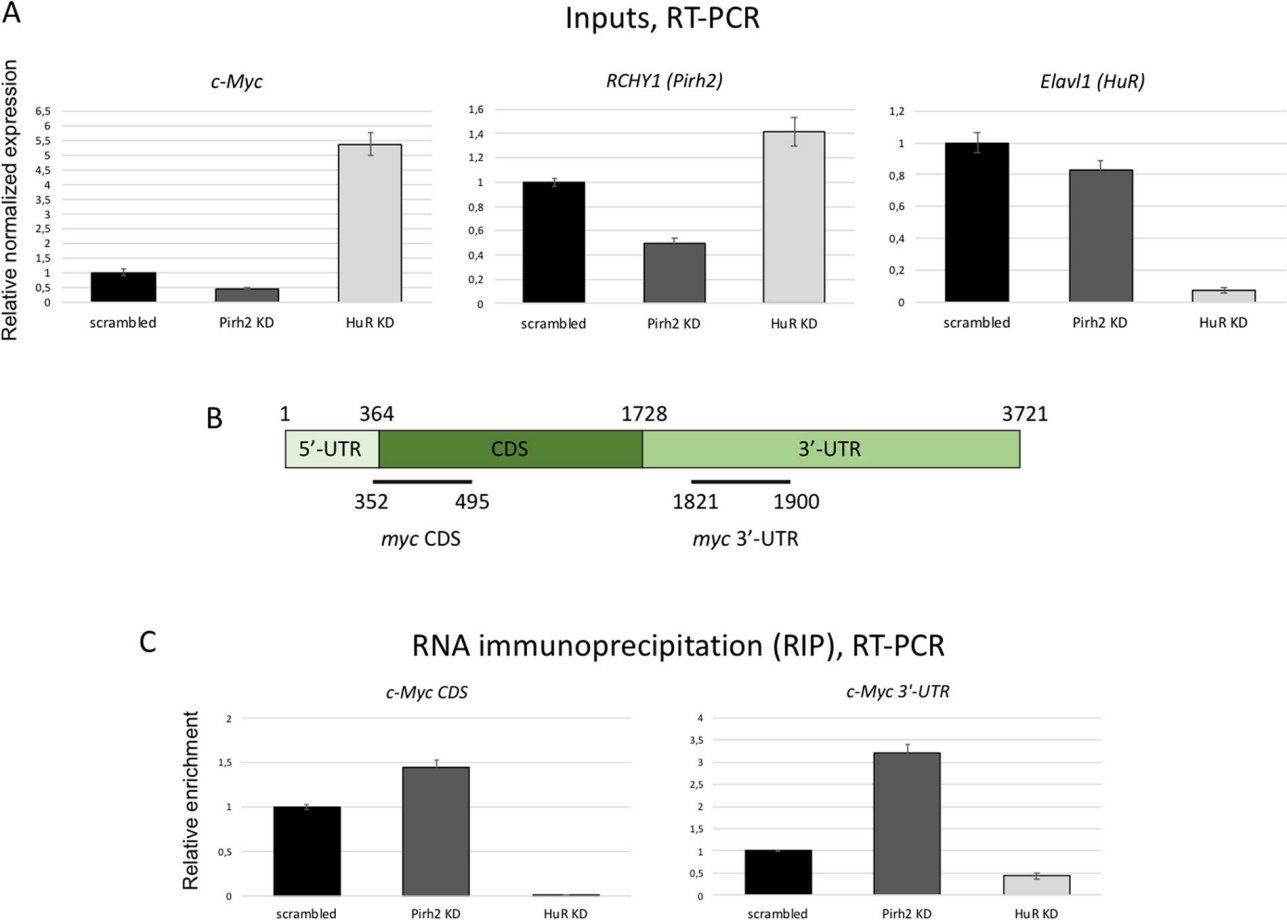
Pirh2 prevents HuR binding to c-Myc mRNA. **A** qRT-PCR demonstrating the expression of c-Myc, HuR, and Pirh2 mRNA in Pirh2 knockdown (Pirh2 KD), HuR knockdown (HuR KD), and control (scrambled) H1299 cells used for RNA immunoprecipitation (RIP assay). **B** The scheme of c-Myc-coding mRNA (NCBI Reference Sequence: NM_002467.6) with indicated regions covered by primes used for RIP assay. CDS, coding sequence; UTR, untranslated regions. **C** RNA immunoprecipitation results obtained by qRT-PCR.

As it was noted above, HuR was shown to downregulate c-Myc via recruiting the RISC complex to its mRNA^42^. To additionally confirm our hypothesis that Pirh2 augments c-Myc levels through downregulation of HuR, we performed RIP assay. We used H1299 cell lines with attenuated expression of either Pirh2 or HuR. H1299 cells with scrambled shRNA were used as control (Fig. 6A). As HuR was reported to preferentially bind 3′-UTRs of its target genes, we used two pairs of primers for RT-PCR analysis specific for the coding region (CDS) and 3′-UTR of c-*myc* (Fig. 6B). We reasoned that the suppression of Pirh2 would increase the cellular level of HuR and hence augment the amount of c-Myc mRNA co-precipitated with HuR. In fact, KD of Pirh2 promoted the binding of c-Myc mRNA to HuR. Importantly, the most pronounced difference in binding was observed in the c-myc 3′-UTR region compared to c-myc CDS (Fig. 6C). These results correlate with the previously published data about the HuR-binding specificity^42^. We used HuR KD cells as control and demonstrated the decreased amounts of c-Myc mRNA precipitation (Fig. 6C). Interestingly, in Pirh2 KD cells we also observed a moderate decrease of HuR mRNA. Thus, we confirmed that the effect of Pirh2 on c-Myc expression is mediated via the Pirh2-dependent regulation of HuR.

### Pirh2 stimulates proliferation of H1299 cell and cooperates with c-Myc in lung cancer progression

To demonstrate the functional effect of Pirh2-dependent attenuation of HuR, we tested the effect of Pirh2 expression on proliferation of H1299 non-small cell lung cancer cells.

**Fig. 7:**
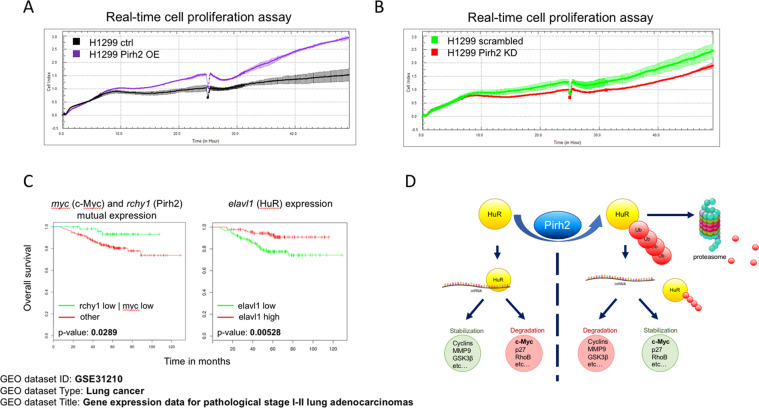
Pirh2 activates H1299 cell proliferation and cooperates with c-Myc in lung cancer progression. **A** Pirh2 stimulates proliferation of H1299 cells. Cell index graphs demonstrating the proliferation registered by xCelligence system for H1299 cells with stable Pirh2 overexpression (Pirh2 OE) and control H1299 cells (ctrl). **B** Pirh2 knockdown suppresses proliferation of H1299 cells. Cell index graphs registered by xCelligence system for H1299 cells with stable Pirh2 knockdown (Pirh2 KD) and control H1299 cells (scrambled). **C** The bioinformatics analysis of the effect of simultaneous *rchy1* and *myc* genes (coding Pirh2 and c-Myc, respectively), and *elavl1* gene (coding HuR protein) expression on lung adenocarcinoma patients’ (GEO dataset ID: GSE31210) survival probability. *P*-values are indicated. **D** Graphical scheme summarizing the role of Pirh2 in HuR protein stability regulation. Pirh2 ubiquitinates HuR that leads to HuR proteasomal degradation and influence on HuR target mRNA stability.

To measure the proliferation rate of H1299 cells with different status of Pirh2, we performed real-time monitoring of cell growth using an xCELLigence system (Fig. 7A, B). This approach is based on the continuous measurement of the impedance difference of metal bottom of the cell chambers upon the cell growth and spreading. Therefore, this system provides the opportunity to monitor the number of cells attached to the bottom well in real time. We noticed that Pirh2 OE increased the proliferation rate of H1299 cells (compare H1299 Pirh2 OE vs. H1299 ctrl) (Fig. 7A). On the contrary, KD of Pirh2 retarded the proliferation rate of cells (H1299 Pirh2 KD vs. H1299 scrambled) (Fig. 7B).

As the proliferation activity is one of the major characteristics of cancer cells, we examined whether Pirh2 and c-Myc expression levels correlate with survival rates of lung cancer patients. Indeed, as shown in Fig. 7C, reduced expression of *Myc* and *RCHY1* genes, positively correlated with the survival of lung cancer patients. On the contrary, high level of HuR (Elavl1) expression within the same GEO dataset correlated with the prolonged survival of cancer patients (Fig. 7D).

Thus, we showed that increased levels of Pirh2 and c-Myc displayed the oncogenic effects using both the cellular model of lung cancer and the bioinformatics approach using the patient-derived gene expression data.

## Discussion

A wealth of data available in the literature strongly suggests that E3 ligase Pirh2 exerts its functions both in p53-dependent and independent ways^43,44^. Furthermore, Pirh2 can play opposite roles in tumorigenesis depending on the cellular context. For example, overexpression of Pirh2 inhibits epithelial to mesenchymal transition by ubiquitinating Twist and sending the latter for proteasome-dependent degradation^45^. Furthermore, Pirh2 is able to attenuate the nuclear factor-κB (NF-κB) pathway in bortezomib-resistant multiple myeloma cells via ubiquitination of pIKBa and IKKa, the two critical regulators of NF-κB^46^. On the other hand, several studies including our own work, have shown that Pirh2 promotes tumorigenesis by degrading p53, p63, p73, Chk2, p27^kip1^, and other important tumor suppressor proteins^3,4,6,15,17^.

Cellular functions of the protein of interest are often governed by its interacting partners^33^. Therefore, it seemed important to identify new interacting partners of Pirh2, to elucidate its physiological role in cells. E3 ligases, including the Pirh2 protein, often interact with their substrates only transiently. Therefore, we chose mild GST-pulldown conditions to capture as many transiently Pirh2-associated proteins as possible. Subsequently, the proteomic approach has identified 225 Pirh2-interacting proteins. Importantly, a large portion of Pirh2 interactors belonged to RNA processing/splicing factors (Fig. 1). Among those, we focused on the RNA-binding protein, HuR (ELAVL1), because the latter has previously been reported to participate in the p53 regulation.

The HuR protein is a product of the *ELAVL1* gene and one of the members of the embryonic lethal abnormal vision (ELAV)/Hu family. In most cases, HuR binds to U- and AU-rich motifs in the 3′-UTR region of mRNAs and differentially affects their stability^47,48^. By regulating the mRNA stability, HuR plays various roles in the cell including its participation in mRNA nuclear cytoplasmic shuttling^49^ and splicing^50,51^. In the cytoplasm, HuR regulates the stability of target mRNAs, both positively and negatively. For example, HuR increases the half-life of several mRNAs, including MMP9, cyclins A, B1, D1, and GSK3β^52–54^. On the contrary, other mRNAs (e.g., p27, RhoB, and c-Myc) are either repressed or degraded by HuR^42,55,56^. Specifically, HuR inhibits the expression of CDKN1B by binding the 5′-UTR to the gene in the region of internal ribosomal entry site (IRES), thereby attenuating the process of CDKN1B translation^56^. On the other hand, HuR mediates degradation of c-Myc mRNA by recruiting RISC loaded with microRNA let-7, which in turn targets the c-Myc 3′-UTR^42^. In this respect, it is important to note that we have previously shown Pirh2-mediated upregulation of c-Myc expression in non-small lung carcinoma cells, both on the level of its mRNA and protein translation^24^.

In line with this observation, in the present study we showed that Pirh2 regulated c-Myc via affecting the stability of HuR by ubiquitinating the latter. In contrast, ablation of Pirh2 caused stabilization of HuR, which in turn decreased the level of c-Myc expression in H1299 cells.

To date, only one E3 ligase,TRIM21, has been reported to ubiquitinate HuR^57^. This TRIM21-dependent ubiquitination caused HuR degradation as part of the UV response and subsequently affected p53 accumulation under UV-induced stress. In addition to that study, we demonstrated the universal Pirh2-dependent mechanism of HuR attenuation, both under normal condition and under cellular stress caused by heat shock or DNA damage.

Taking into account the number of HuR mRNA targets playing central roles in key cellular processes, it is not surprising that HuR expression is shown to be a biomarker for numerous cancers^58–60^. In particular, elevated cytoplasmic HuR presence was shown to be a marker of poor prognosis and cancer aggressiveness for bladder cancer, meningioma, lung cancer, and esophageal squamous cell carcinoma^61–64^. However, high expression levels of HuR may also have a favorable prognostic value, e.g., for breast cancer^65^, which may be associated with HuR-mediated stabilization of p53 and hence activation of its proapoptotic downstream targets^66–68^.

Using the bioinformatics approach, we revealed that low Pirh2 and c-Myc levels and high HuR level correlated with better survival prognosis of patients with lung adenocarcinoma (Fig. 7C). To corroborate our results, we propose a model (Fig. 7E), whereby Pirh2 attenuates the protein stability of HuR and hence modulates levels of expression of HuR target genes. Future studies should reveal the effect of Pirh2 on other mRNA targets of HuR.

## Supplementary information

Suppementary Figure S1

Suppementary Figure S2

Suppementary Figure S3

Suppementary Figure S4

Suppementary Figure S5

Suppementary Figure S6

Suppementary Figure S7

Suppementary Figure S8

Supplementary Table (xlsx)

Supplementary figure and table legends
