# Supplementary figures and images for: The RNA-binding protein HuR is a novel target of Pirh2 E3 ubiquitin ligase
Source: Cell Death Dis. 2021 Jun 5;12(6):581. doi: 10.1038/s41419-021-03871-w (PMC8179929; doi:10.1038/s41419-021-03871-w)

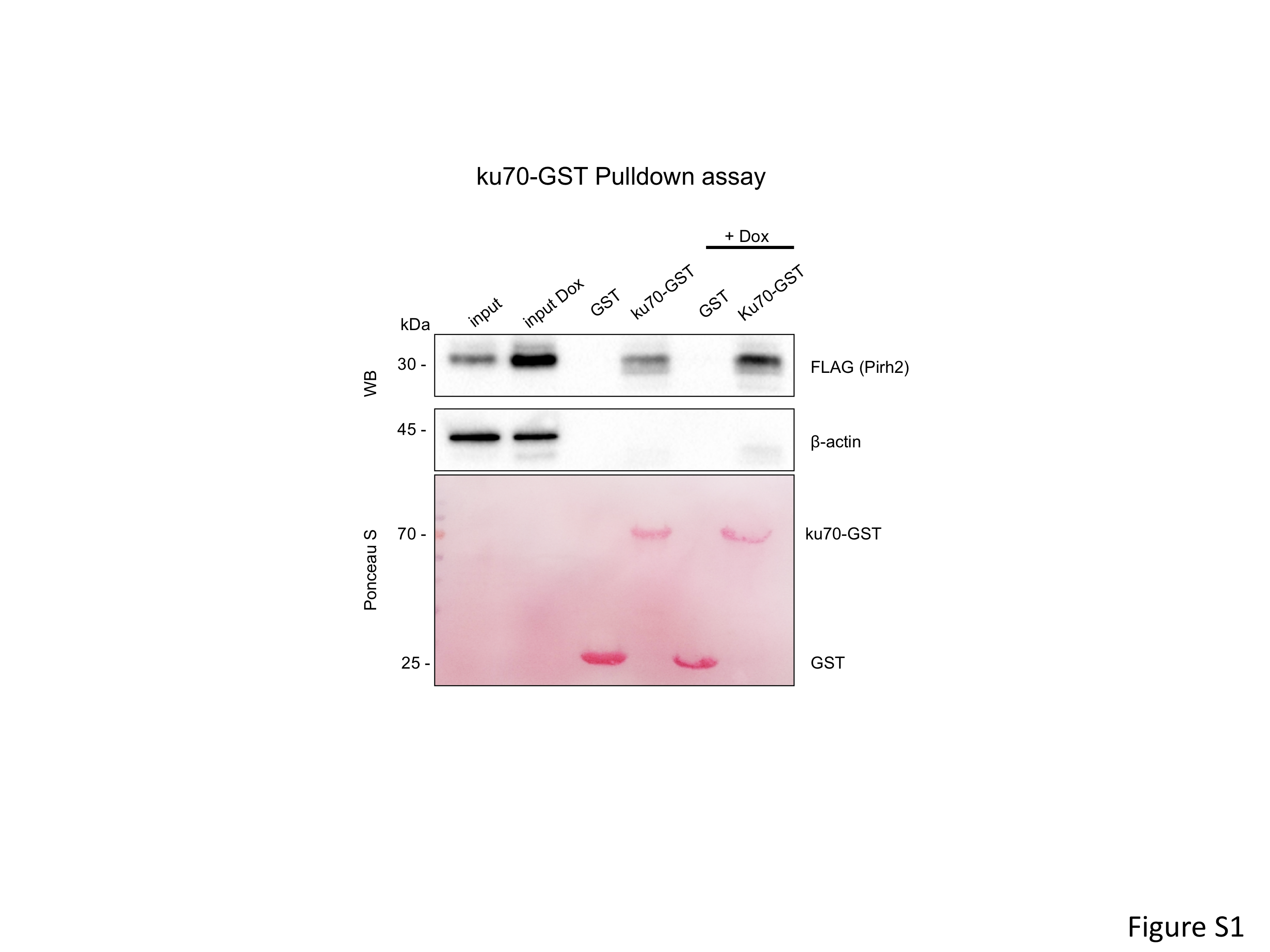

Supplement: Supplementary file 1 — Suppementary Figure S1 [file 41419_2021_3871_MOESM1_ESM.tif]

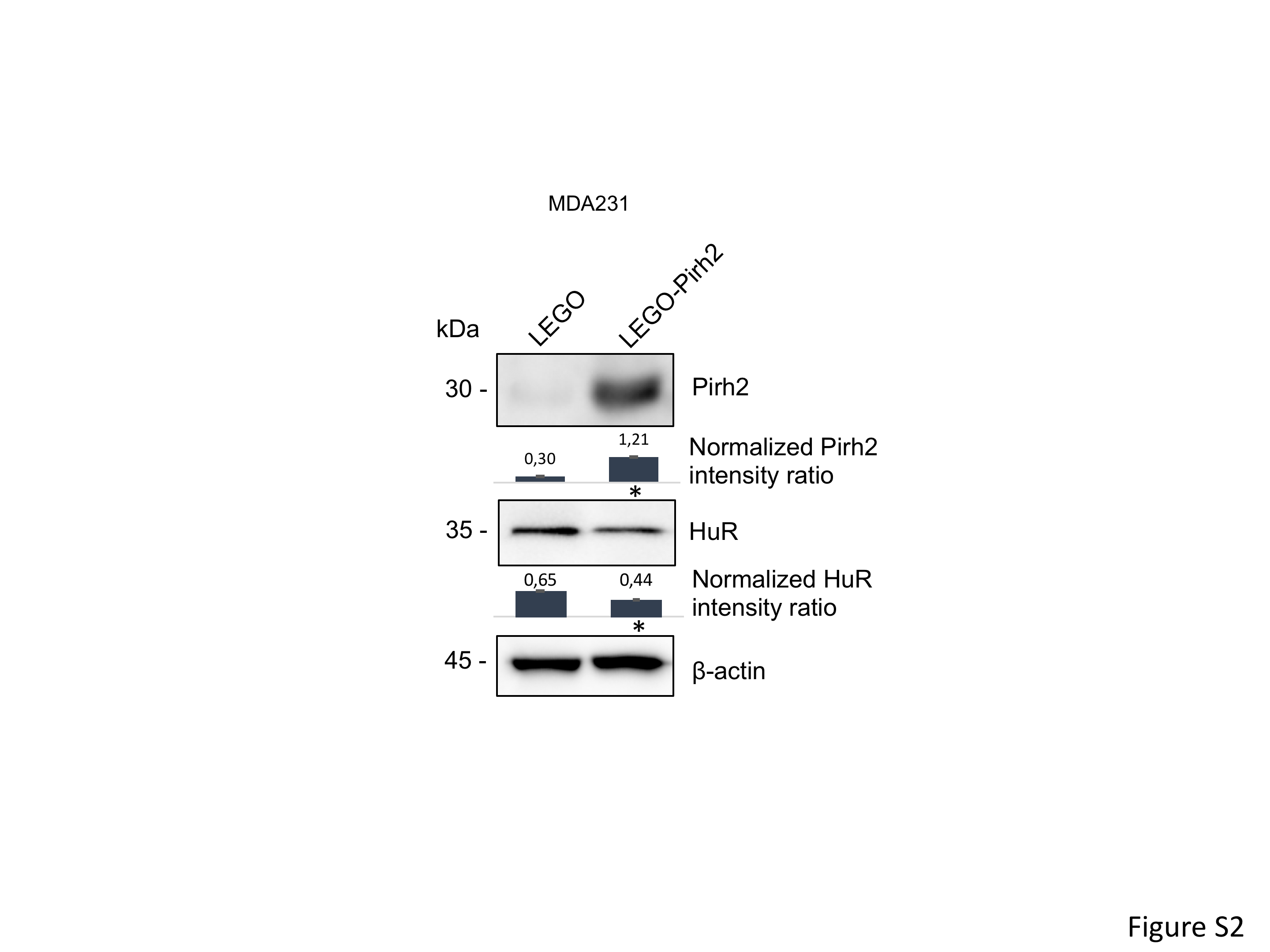

Supplement: Supplementary file 2 — Suppementary Figure S2 [file 41419_2021_3871_MOESM2_ESM.tif]

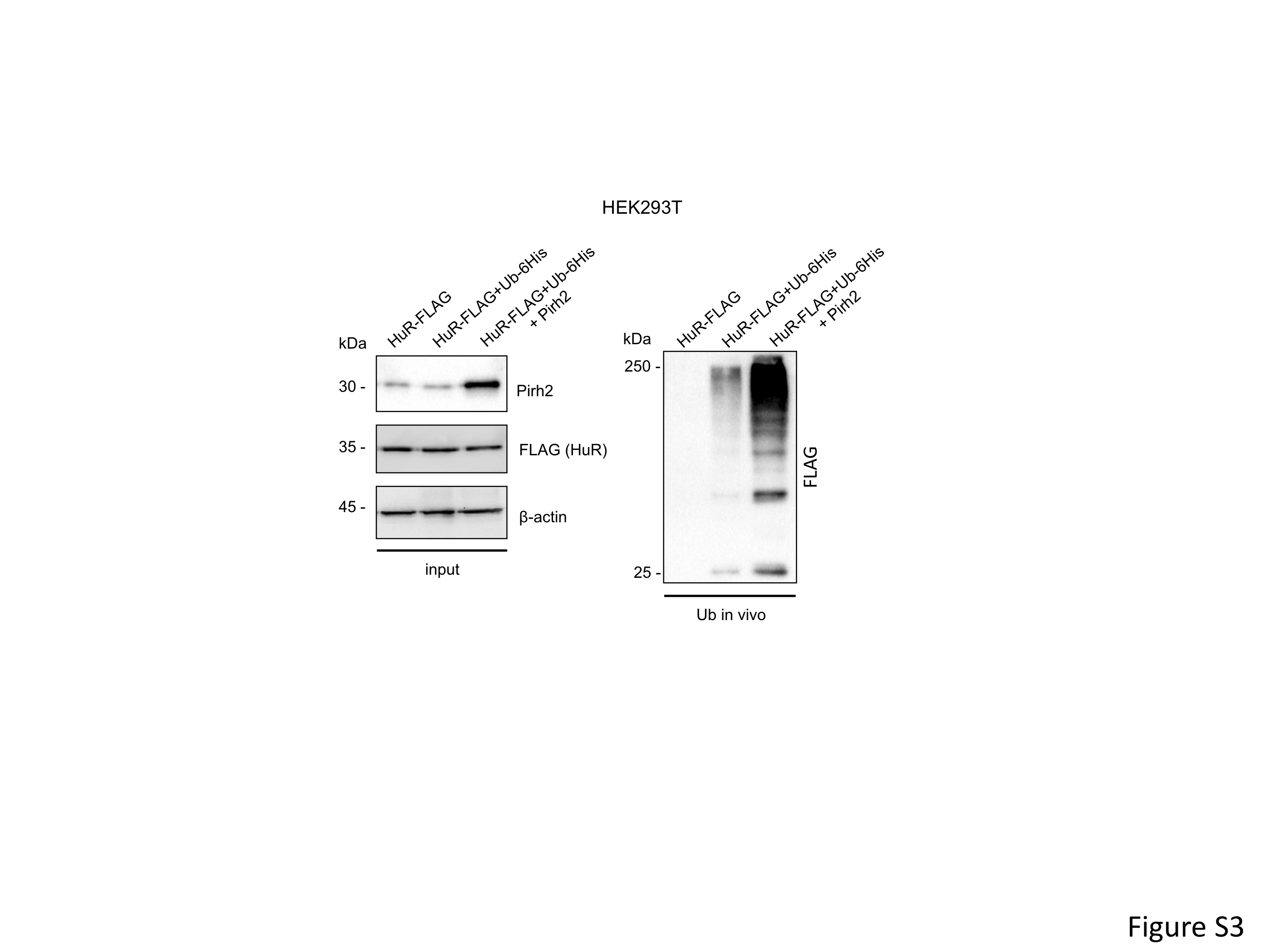

Supplement: Supplementary file 3 — Suppementary Figure S3 [file 41419_2021_3871_MOESM3_ESM.tif]

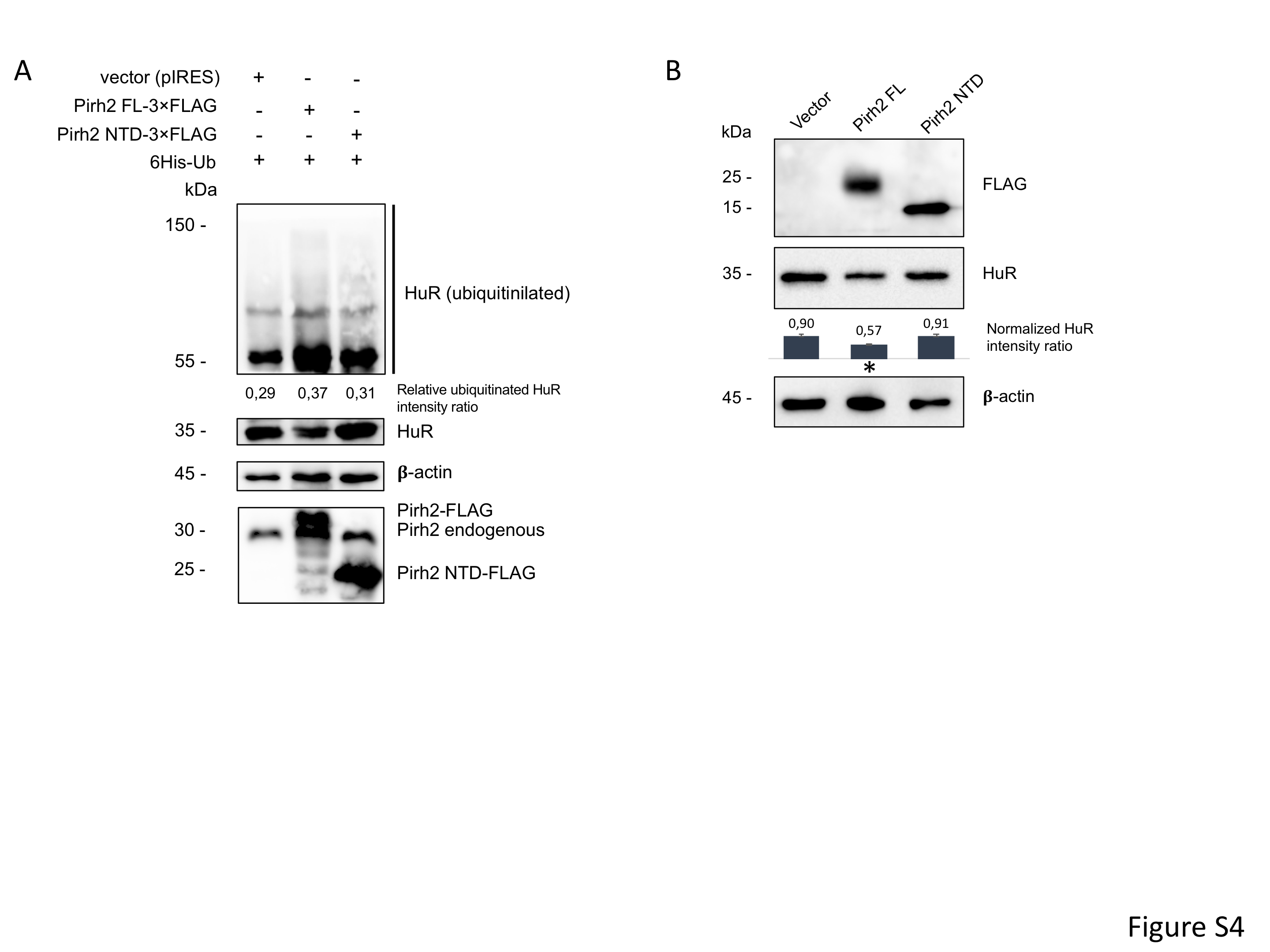

Supplement: Supplementary file 4 — Suppementary Figure S4 [file 41419_2021_3871_MOESM4_ESM.tif]

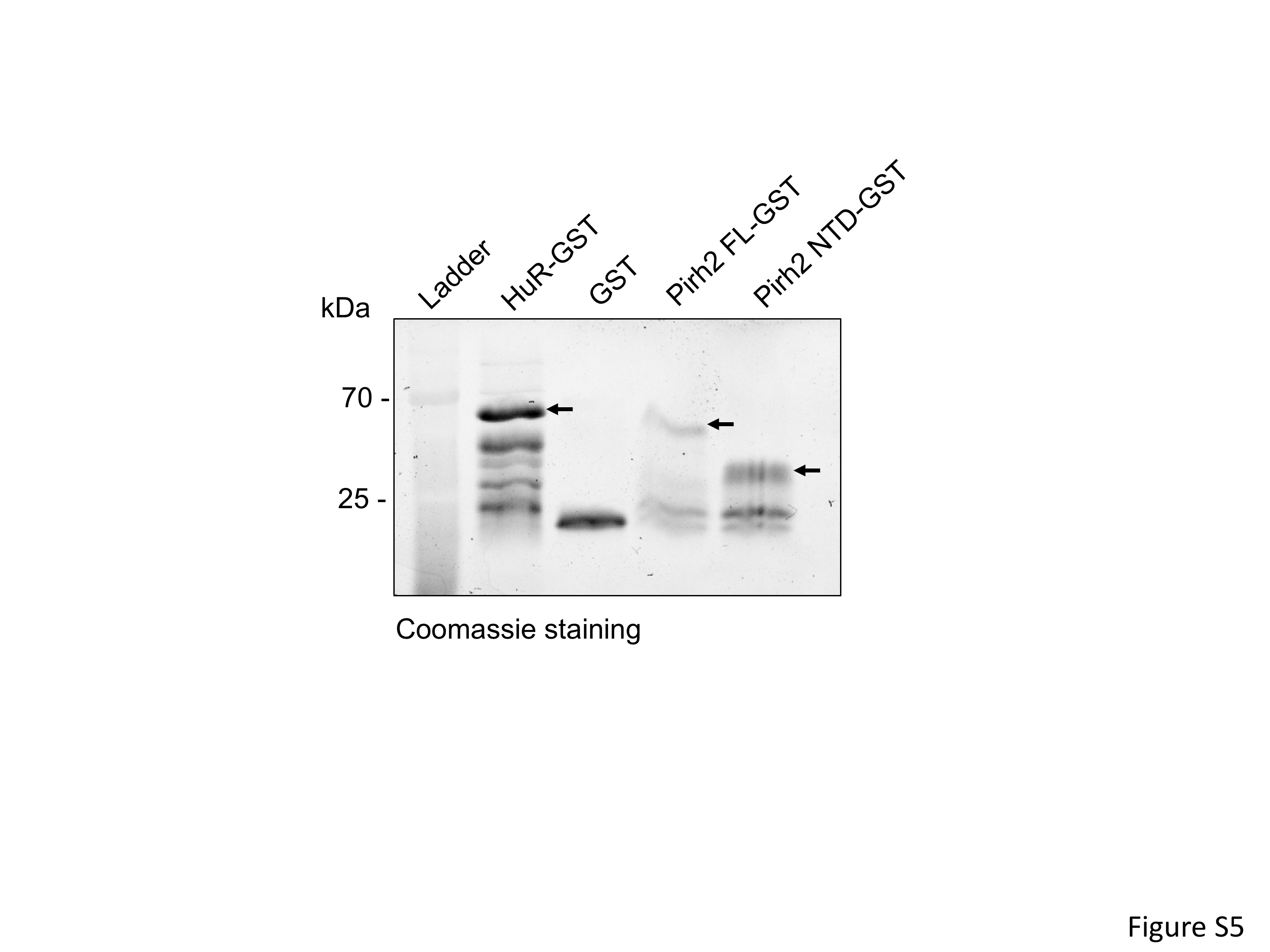

Supplement: Supplementary file 5 — Suppementary Figure S5 [file 41419_2021_3871_MOESM5_ESM.tif]

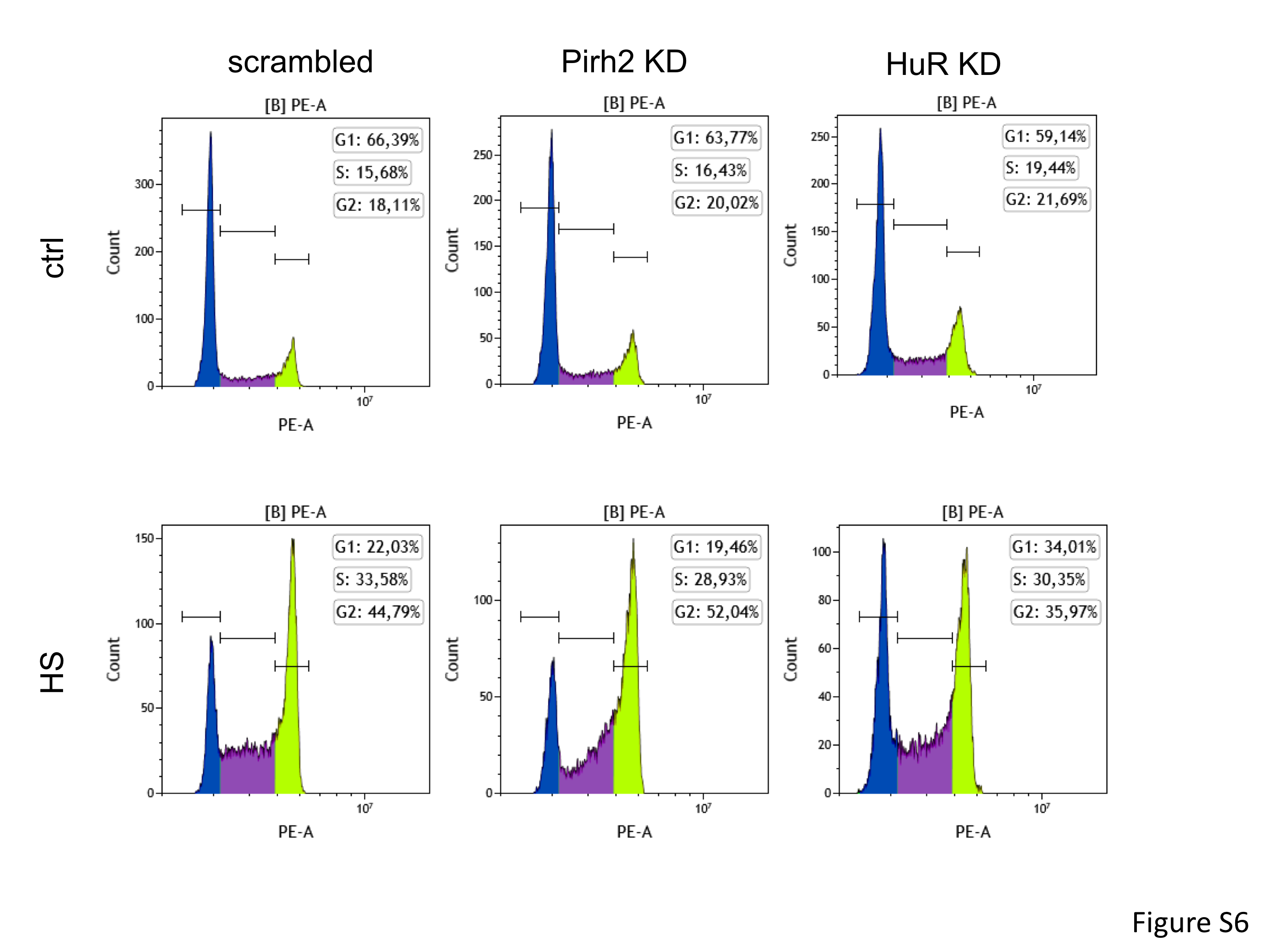

Supplement: Supplementary file 6 — Suppementary Figure S6 [file 41419_2021_3871_MOESM6_ESM.tif]

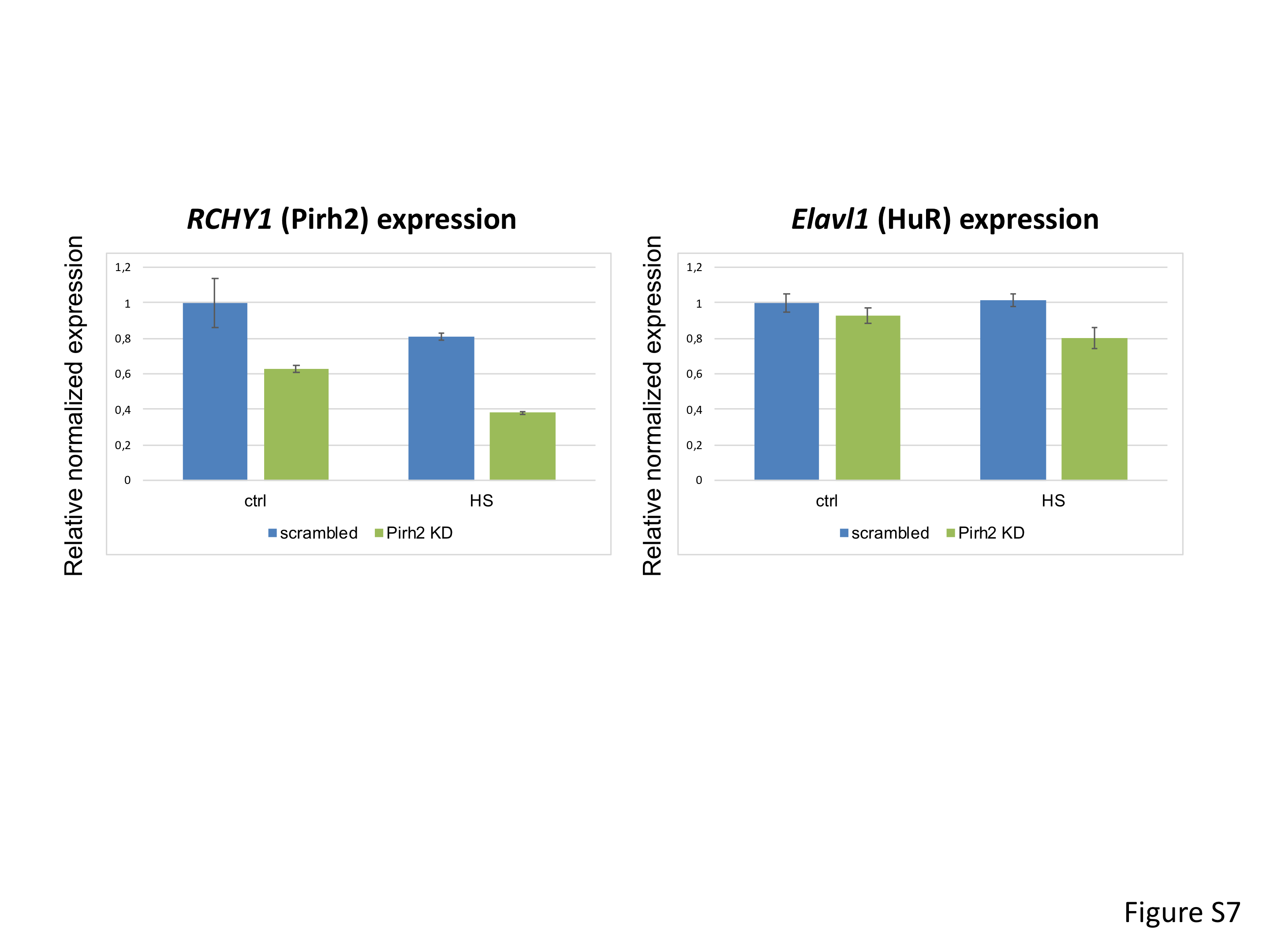

Supplement: Supplementary file 7 — Suppementary Figure S7 [file 41419_2021_3871_MOESM7_ESM.tif]

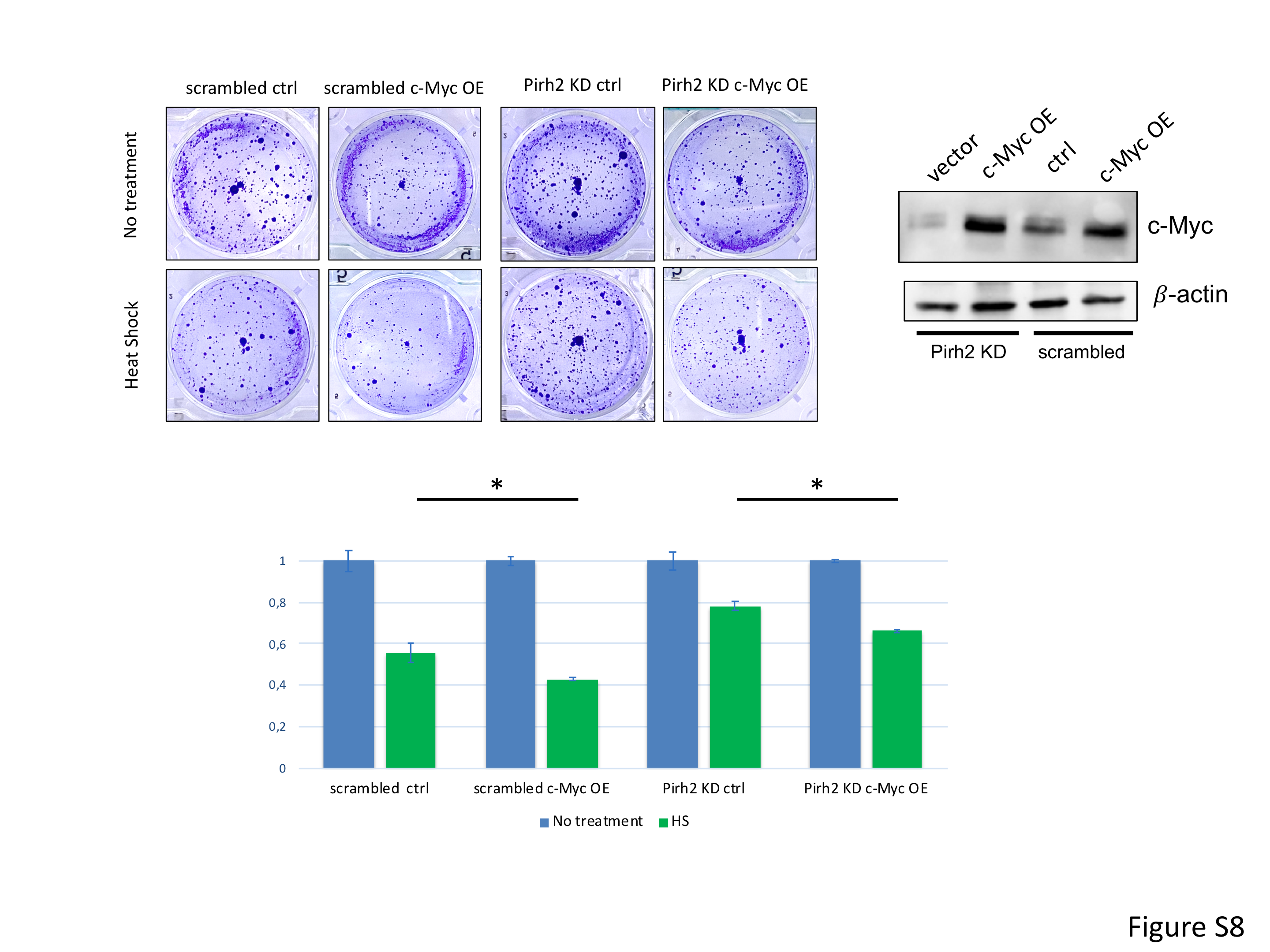

Supplement: Supplementary file 8 — Suppementary Figure S8 [file 41419_2021_3871_MOESM8_ESM.tif]
