# Supplementary material for: The RNA-binding protein HuR is a novel target of Pirh2 E3 ubiquitin ligase
Source: Cell Death Dis. 2021 Jun 5;12(6):581. doi: 10.1038/s41419-021-03871-w (PMC8179929; doi:10.1038/s41419-021-03871-w)
Supplement: Supplementary file 10 — Supplementary figure and table legends [file 41419_2021_3871_MOESM10_ESM.docx]

**SUPPLEMENTARY FIGURES AND TABLE LEGEND**

**Supplementary figure S1**

The Ku70 protein interacts with Pirh2. GST-pulldown assay with extracts from HEK293T cells transfected with the Pirh2-3×FLAG-coding plasmid. The Ku70-GST-Pirh2 interactions both in normal conditions and after 0,5 μM doxorubicin treatment for 24 h (Dox) are shown. Western blot analysis of Pirh2 binding to GST and ku70-GST (upper panel) was analyzed using anti-Pirh2 antibody; a Ponceau-stained membrane is shown as loading control (lower panel).

**Supplementary figure S2**

Western blot analysis of the HuR levels in MDA-MB-231cells stably expressing LEGO-Pirh2 versus control MDA-MB-231 LEGO cells. The normalized intensity ratios were calculated as ratios between the signals of proteins analyzed and the corresponding actin bands on the basis of three measurements. Error bars indicate ±SD. * indicates p ≤0.05 versus LEGO according to Student's t-test.

**Supplementary figure S3**

(Left) Western blot analysis of protein levels for Pirh2, HuR (FLAG), and actin (loading control) in Input lanes. (Right) Ubiquitination levels of the HuR protein purified on Ni2+ NTA from HEK293T cells transfected with plasmids indicated on the top.

**Supplementary figure S4**

**A:** Western blot analysis of ubiquitinated endogenous HuR protein from HeLa cells transfected with 6His-Ubiquitin and plasmids indicated on the top. The normalized intensity ratio was calculated as ratio between the signals of Ubiquitinated HuR and the corresponding actin bands. **B:** Western blot analysis of HuR levels in HeLa cells transiently transfected with 3×FLAG-tagged full-length Pirh2 (Pirh2 FL), the deletion mutant of Pirh2 (Pirh2 NTD) and an empty vector as negative control. The normalized intensity ratios were calculated as ratios between the signals of proteins analyzed and the corresponding actin bands on the basis of three measurements. Error bars indicate ±SD. * indicates p ≤0.05 versus empty vector according to Student's t-test.

**Supplementary figure S5**

The Coomassie-stained gel demonstrating the amounts of purified proteins: HuR-GST, GST, Pirh2-GST and the deletion mutant Pirh2-GST NTD used in the *in vitro* ubiquitination reaction.

**Supplementary figure S6**

The cell cycle distribution in Pirh2 knock-down (Pirh2 KD), HuR knock-down (HuR KD), and control (scrambled) HeLa cells in normal condition (ctrl), and after HS (45°C for 2 h) followed by 16 h recovery (HS).

**Supplementary figure S7**

The effect of Pirh2 knock-down in HeLa cells on the levels of HuR
expression in HeLa cells both in normal condition and after HS (43°C for 120 minutes) measured of qRT-PCR.

**Supplementary figure S8**

The effect of c-Myc transient over-expression on the ability of Pirh2KD or scrambled shRNA HeLa cells to form colonies after the heat shock treatment. The relative colony counts are provided. The test was performed in triplicates. Error bars indicate ±SD. * indicates p-value ≤0.01 according to Student's t-test.

**Supplementary Table**

Supplementary table contains processed LC-MS/MS results data excluding prokaryotic proteins, keratins, Pirh2 protein and glutathione-S-transferase from the list. Each identified protein, for which at least 3 peptides were identified (score ≥ 3), and which either does not bind GST, or binds it to a much lesser extent than the Pirh2-GTS, was defined by us as significant. After processing the data obtained, we revealed 225 Pirh2-interacting proteins. The row data are publicly accessible on the Mendeley repository (DOI: 10.17632/24bh4cvhzd.1).
